# Multi-patient dose synthesis of [^18^F]Flumazenil via a copper-mediated ^18^F-fluorination

**DOI:** 10.1186/s41181-022-00158-z

**Published:** 2022-03-20

**Authors:** Thibault Gendron, Gianluca Destro, Natan J. W. Straathof, Jeroen B. I. Sap, Florian Guibbal, Charles Vriamont, Claire Caygill, John R. Atack, Andrew J. Watkins, Christopher Marshall, Rebekka Hueting, Corentin Warnier, Véronique Gouverneur, Matthew Tredwell

**Affiliations:** 1 Trasis, Rue Gilles Magnée, 90, 4430 Ans, Belgium; 2 Chemistry Research Laboratory, University of Oxford, 12 Mansfield Road, Oxford, OX1 3TA UK; 3 Medicines Discovery Institute, Cardiff University, Main Building, Park Place, Cardiff, CF10 3AT UK; 4 Wales Research and Diagnostic PET Imaging Centre, Cardiff University, University Hospital of Wales, Heath Park, Cardiff, CF14 4XN UK; 5 School of Chemistry, Cardiff University, Main Building, Park Place, Cardiff, CF10 3AT UK

**Keywords:** [^18^F]Flumazenil, Automation, Fluorine-18, Good manufacturing practice, Positron emission tomography

## Abstract

**Background:**

Flumazenil (FMZ) is a functionally silent imidazobenzodiazepine which binds to the benzodiazepine binding site of approximately 75% of the brain γ-aminobutyric acid-A receptors (GABA_A_Rs). Positron Emission Tomography (PET) imaging of the GABAARs with [^11^C]FMZ has been used to evidence alterations in neuronal density, to assess target engagement of novel pharmacological agents, and to study disorders such as epilepsy and Huntington’s disease. Despite the potential of FMZ PET imaging the short half-life (*t*_1/2_) of carbon-11 (20 min) has limited the more widespread clinical use of [^11^C]FMZ. The fluorine-18 (^18^F) isotopologue with a longer *t*_1/2_ (110 min) is ideally suited to address this drawback. However, the majority of current radiochemical methods for the synthesis of [^18^F]FMZ are non-trivial and low yielding. We report a robust, automated protocol that is good manufacturing practice (GMP) compatible, and yields multi-patient doses of [^18^F]FMZ.

**Results:**

The fully automated synthesis was developed on the Trasis AllinOne (AIO) platform using a single-use cassette. [^18^F]FMZ was synthesized in a one-step procedure from [^18^F]fluoride, via a copper-mediated ^18^F-fluorination of a boronate ester precursor. Purification was performed by semi-preparative radio-HPLC and the collected fraction formulated directly into the final product vial. The overall process from start of synthesis to delivery of product is approximately 55 min. Starting with an initial activity of 23.6 ± 5.8 GBq (*n* = 3) activity yields of [^18^F]FMZ were 8.0 ± 1 GBq (*n* = 3). The synthesis was successfully reproduced at two independent sites, where the product passed quality control release criteria in line with the European Pharmacopoeia standards and ICH Q3D(R1) guidelines to be suitable for human use.

**Conclusion:**

Reported is a fully automated cassette-based synthesis of [^18^F]FMZ that is Good Manufacturing Practice (GMP) compatible and produces multi-patient doses of [^18^F]FMZ.

**Supplementary Information:**

The online version contains supplementary material available at 10.1186/s41181-022-00158-z.

## Background

Positron emission tomography (PET) imaging of the γ-aminobutyric acid-A (GABA_A_) receptors with [^11^C]flumazenil (FMZ) is effective for assessing target engagement of novel pharmacological agents of the benzodiazepine recognition site (Atack et al. 2010; Eng et al. 2010), and for the localization and determination of the extent of epileptic foci in patients with temporal lobe epilepsy (Savic et al. 1988; Koepp et al. 1997). A comparative clinical study concluded that pre-surgical [^11^C]FMZ imaging provides complementary data to [^18^F]fluoro-2-deoxy-D-glucose ([^18^F]FDG) and MRI in the localization of epileptogenic foci, and may correlate more precisely with the anatomical extent of the abnormality in comparison to [^18^F]FDG PET (Ryvlin et al. 2020). The relatively short half-life of carbon-11 (*t*_1/2_ = 20 min) prevents the broader clinical use and study of this radiotracer for epilepsy, as well as other fields such as neurodegeneration, stroke and anxiety disorders (Holthoff et al. 1993; Heiss et al. 2000; Malizia et al. 1998).

**Fig. 1:**
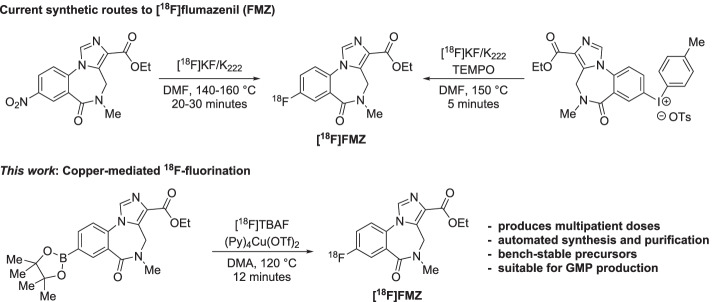
Existing protocols and new copper-mediated ^18^F-fluorination approach

As flumazenil contains an aryl fluoride motif it is possible to use the isotopologue with the PET radionuclide fluorine-18 (^18^F) without any chemical modification to the parent structure (Vivash et al. 2013). With a half-life of 110 min, fluorine-18 radiotracers are advantageous over carbon-11 by virtue of allowing for the imaging of multiple patients from a single radiotracer production, as well as delivery of the fluorine-18 radiotracers off-site. Despite the advantages of [^18^F]FMZ over [^11^C]FMZ, the widespread use of the former has been hampered by low radiochemical yields (RCY), or inaccessibility of requisite precursors. The synthesis of [^18^F]FMZ from nitromazenil and [^18^F]fluoride is hindered by the relatively unactivated nature of the arene (Ryzhikov et al. 2005; Schirrmacher et al. 2012; Kumar et al. 2021), and further compounded by the non-trivial separation of the nitromazenil precursor from [^18^F]FMZ (Massaweh et al. 2009; Vaulina et al. 2018). An alternative strategy towards [^18^F]FMZ has featured a diaryliodonium precursor, giving improved radiochemical yields, and was used to support clinical studies. This latter method has not been widely implemented for routine production presumably due the poor stability and availability of the iodonium precursors (Moon et al. 2011; Moon et al. 2014) (Fig. 1).

In recent years several new radiochemical methods have emerged for the synthesis of ^18^F-fluoro arenes from [^18^F]fluoride (Brooks et al. 2014; Preshlock et al. 2016a; Deng et al. 2019). Of these methods the copper-mediated ^18^F-fluorination of aryl boronic species, reported by Gouverneur and co-workers (Tredwell et al. 2014), has been applied to a wide variety of structurally diverse targets, including FMZ (Wright et al. 2020). Clinical translation of this copper-mediated technology to a fully automated process has been challenging, largely due to the detrimental effect on the RCYs of the basic solutions used to elute [^18^F]fluoride from ion-exchange cartridges (Mossine et al. 2017; Zlatopolskiy et al. 2015). Significant progress has been made to address these challenges as highlighted by the recent reports on the automated synthesis of [^18^F]olaparib (Guibbal et al. 2020) and 6-[^18^F]fluoro-ʟ-DOPA (Mossine et al. 2020). In the context of these results, and based on our previous work (Preshlock et al. 2016b), we reasoned that this copper-mediated ^18^F-fluorination method would be well-suited to solving the long-standing issues associated with [^18^F]FMZ synthesis.

Our goals were to develop a “plug-and-play” single use cassette-based system that could produce multi-patient doses, and only use chemical reagents that are easily analysed using existing quality control (QC) protocols to facilitate the implementation and validation of this method for use in PET centres.

## Methods

### General

All reagents and solvents were purchased from Merck Life Science. Flumazenil (FMZ) was purchased from Tokyo Chemical Industry (TCI) UK. Flumazenil impurity B (OHMZ) was purchased from Merck Life Science UK. Ethyl 5-methyl-6-oxo-8(4,4,5,5-tetramethyl-1,3,2-dioxaborolan-2-yl)-5,6-dihydro-4H-benzo[f]imidazo[1,5-a][1,4]diazepine-3-carboxylate (FMZ-BPin) and ethyl 5-methyl-6-oxo-5,6-dihydro-4H-benzo[f]imidazo[1,5-a][1,4]diazepine-3-carboxylate (HMZ) were synthesised as described previously (Preshlock et al. 2016b; Gu et al. 1993). Solid phase extraction (SPE) and QMA carbonate cartridges were purchased from Waters. Quantofix® copper-specific test strips were purchased from Macherey Nagel. Sterile 0.9% saline solution bottles were obtained from Baxter. Automated radiochemical syntheses were performed on a Trasis AllinOne (AIO) equipped with a Phenomenex Luna® 5 μm C18(2) 100 Å column (10 × 250 mm). Fluid pathways, consumables, and tetrabutylammonium bicarbonate (TBA-HCO_3_) solution were supplied by Trasis.

The methods of radiochemical production and analysis are respectively described at the two sites; [^18^F]fluoride was produced using the ^18^O(p,n)^18^F reaction. Radio-thin layer chromatography (radio-TLC) was performed on aluminum sheets coated with silica gel 60 F_254_ (Macherey Nagel) and analyzed with a LabLogic Scan-RAM radio-TLC scanner equipped with PS detector, or radio-TLC scanner (Bioscan AR-2000). Radio-high-performance liquid (radio-HPLC) chromatography was performed on an Agilent 1200 equipped with a LabLogic gamma-RAM Model 4 detector, or on an Alliance system (Waters) equipped with a GABI Star NaI(Tl) scintillation detector (energy window 400–700 keV, Elysia-Raytest). Gas chromatography (GC) was performed on a PerkinElmer Clarus 500 with a TurboMatrix 40 headspace sampler fitted with a Restek Rxi-624Sil column, or Shimadzu GC-2010 system with flame ionization detection. The pH of aqueous solutions were measured using a Mettler-Toledo SevenMulti pH meter, or a Mettler-Toledo Five-Easy pH meter, calibrated at pH 4.0, 7.0 and 10.0 with correction for the temperature.

### Fluid path

**Fig. 2:**
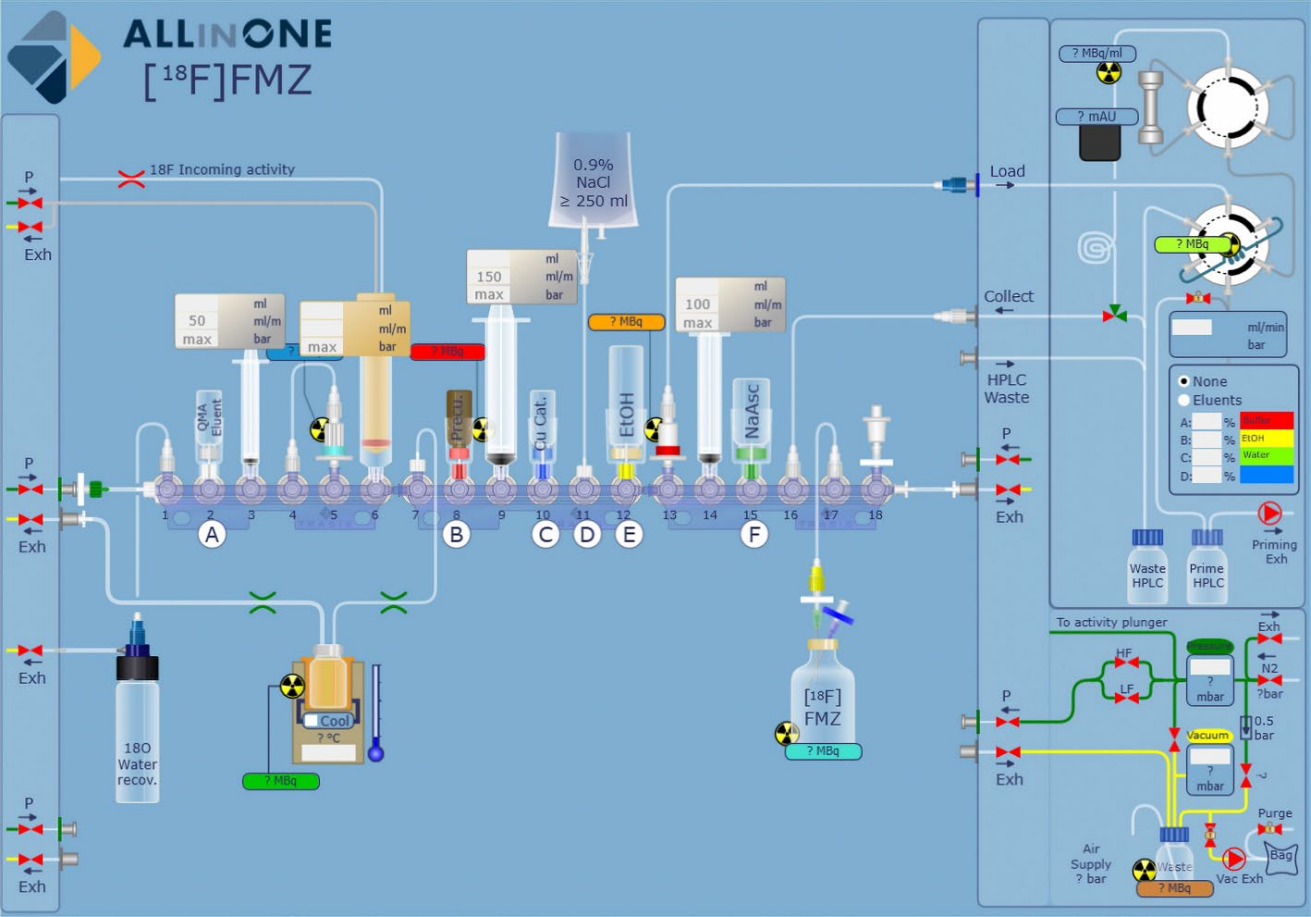
Cassette layout

The AIO synthesizer relies on the use of a disposable fluid path (cassette) to which reagent vials, syringes and reactors are connected via rotary actuated valves. The cassette used in this study was composed of three manifolds of six valves each, one reactor, three syringes (3 mL, 20 mL and 10 mL, respectively), a low bioburden tubing set, and a 20 mL syringe cylinder as incoming activity reservoir (Fig. 2). Each of the two gas-supply ports were equipped with 0.22 µm PTFE filters (Sartorius).

### Radiochemistry

At the start of the synthesis, [^18^F]fluoride was extracted from the ^18^O-enriched-water solution from the cyclotron using an anion exchange cartridge (QMA). The radioactivity was released to the reactor with a solution of tetrabutylammonium bicarbonate in water/acetonitrile. After azeotropic drying of the [^18^F]fluoride mixture, FMZ-BPin (12 mg, 30 µmol) in *N*,*N*-dimethylacetamide (DMA) (500 µl), and tetrakis(pyridine)copper(II) triflate ((Pyr)_4_Cu(OTf)_2_) (27 mg, 40 µmol) in DMA (500 µl), were subsequently added into the reactor. The resulting deep-blue solution was briefly aerated before the system was sealed off. The reaction was then heated at 120 °C for 12 min. After being actively cooled to 50 °C, the reaction was quenched with saline and the resulting solution was passed through a SepPak tC18 Plus short cartridge in a pre-HPLC purification step. [^18^F]FMZ was released from the cartridge with 23% aqueous ethanol into the HPLC loop. Semi-preparative HPLC was performed with 25% EtOH in 10 mM phosphate buffer (pH 7.2) on a Luna 5 μm C18 (2) column (100 Å, 10 × 250 mm). The collected [^18^F]flumazenil was formulated with 0.9% NaCl and an aqueous solution of sodium ʟ-ascorbate. The total synthesis time was 55 min. Information on automation, cassette assembly, reagents and radiochemical methods are provided in the Additional file 1.

### Quality control

Acceptance criteria were set according to the European Pharmacopoeia and ICH Q3D(R1) guidelines. Colour and turbidity of the formulated solution were assessed visually. pH was measured using a pH-meter. Radiochemical purity was determined by radio-TLC using a 5% methanol in dichloromethane solution as eluent or 100% ethyl acetate. The chemical purity was measured by quantitative radio-HPLC on a Chromolith® Performance RP-18 (100 × 4.6 mm) column with spectrophotometer (246 nm) and radioactivity detectors connected in series. Identity of the radiochemical product and of known impurities were confirmed by coelution with the non-radioactive analogues. Residual solvent analysis was performed by GC on an SH-Stabilwax (30 m × 0.25 mm) column with 1-propanol as internal standard or PerkinElmer Clarus 500 with a TurboMatrix 40 headspace sampler. Residual copper content was qualitatively assessed using dedicated Quantofix® test strips after cross-validation of the method by inductively coupled plasma mass spectrometry (ICP-MS). Osmolality testing could not be performed due to ethanol content of the final formulation.

### In-vitro studies

FMZ, OHMZ, HMZ and PF-06372865 (Nickolls et al. 2018) were all dissolved in dimethyl sulfoxide (DMSO) at a concentration of 10 µM. These samples were serially diluted in 100% DMSO in half-log units such that when 5 µl were added into a total assay volume of 500 µl, the final DMSO concentration in the assay was 1%. Compounds were generally tested over a concentration range in the assay of 0.1 nM to 1 µM.

The affinity of FMZ, OHMZ, HMZ and PF-06372865 for human recombinant GABA_A_Rs were measured using a [^3^H]flumazenil in vitro binding assay as described previously elsewhere (Atack et al. 2006). In brief, mouse fibroblasts stably expressing the four subtypes of human GABA_A_Rs that contain a benzodiazepine binding site (i.e., α1-, α2-, α3- and α5β3γ2) were grown, harvested, and membranes were prepared and frozen at -80 °C until required. On the day of the assay, membranes were thawed and reconstituted in assay buffer (75 mM Tris.Cl, pH 7.4, 1 mM EDTA, 12.5 mM MgCl_2_) at a concentration of 101 µg/ml for α1β3γ2 and 51 µg/ml for α2-, α3- and α5β3γ2 membrane. A mixture of 395 µl of prepared membranes, 100 µl of 40 nM [^3^H]flumazenil (giving a final concentration in the assay of 4 nM) and 5 µl of test compound were incubated together to define the levels of total and non-specific binding, respectively. Incubations were performed for 1 h at room temperature before being terminated by filtration and rapid washing (2 × 10 ml of ice-cold 50 mM Tris.Cl, pH 7.4 buffer) over Whatman GF/B filters using a Brandel cell harvester. Filters were then placed in scintillation vials, 4 ml of scintillation fluid added, and radioactivity was counted on a Tricarb 2900 scintillation counter.

The extent to which the level of specific binding could be inhibited by different concentrations of each compound was calculated and the concentration which inhibited the specific binding by 50% (the IC_50_) was determined. From these values, the Ki for each compound at each different subtype was calculated using the Cheng-Prusoff equation and KD values for [^3^H]flumazenil of 4.57, 6.06, 7.86 and 1.49 nM at α1-, α2-, α3- and α5β3γ2 human GABA_A_Rs, respectively.

## Results

### Automated radiochemical synthesis

**Fig. 3:**
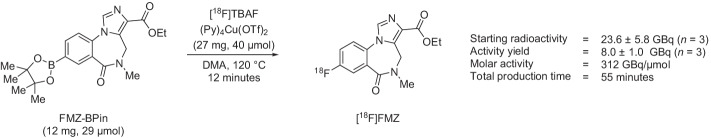
Optimised automation conditions for the production of [^18^F]FMZ

The automated protocol was performed at both sites to assess any variability. Starting with 300–340 MBq (*n* = 5, Trasis) of [^18^F]fluoride this method yielded [^18^F]FMZ in 43 ± 2% n.d.c. Using increased starting activities 23.6 ± 5.8 GBq (*n* = 3, Cardiff) of [18F]fluoride this method yielded [^18^F]FMZ in 35 ± 5% n.d.c. The molar activity of [^18^F]FMZ was measured at 312 GBq/µmol n.d.c. (Fig. 3). In addition to the desired product, we observed the formation of OHMZ (3.7 ± 0.8 µg/V and ≤ 1.5 µg/V) (Flumazenil, European Pharmacopeia, 10.7; 2655), by analytical HPLC by comparison with authentic samples. The protodeborylated compound, HMZ, was not observed as an impurity (Table 1).

**Table 1:** Acceptance criteria and analytical data

Control	Method	Acceptance criteria	Trasis Results (*n* = 5)	Cardiff Results (*n* = 3)
RCY (n.d.c)	Ionization chamber	N/A	43 ± 2%	35 ± 5%
Appearance	Organoleptic	Clear and colorless	Clear and colorless	Clear and colorless
pH	pH-meter	4.5 to 8.5	7.07 ± 0.03	7.5
Chemical purity	HPLC	[^19^F]FMZ ≤ 50 µg/V	[^19^F]FMZ 1.1 ± 0.4 µg/V OHMZ 3.7 ± 0.8 µg/V	[^19^F]FMZ ≤ 1.5 µg/V OHMZ ≤ 1.5 µg/V
	Spot test	TBA-HCO_3_ ≤ 2.6 mg/V	TBA-HCO_3_ ≤ 1.3 mg/V	TBA-HCO_3_ ≤ 2.6 mg/V
	Test strips	Copper (as Cu^2+^) ≤ 340 µg/V	Copper (as Cu^2+^) ≤ 100 µg/V	Copper (as Cu^2+^) ≤ 100 µg/V
Radiochemical purity	Radio-TLC	[^18^F]flumazenil ≥ 95% [^18^F]fluoride ≤ 5%	[^18^F]flumazenil ≥ 98% [^18^F]fluoride ≤ 2%	[^18^F]flumazenil ≥ 99% [^18^F]fluoride ≤ 1%
	Radio-HPLC	[^18^F]flumazenil ≥ 99%	[^18^F]flumazenil > 99%	[^18^F]flumazenil > 99%
Residual solvent	GC	DMA ≤ 1090 ppm Acetonitrile ≤ 410 ppm Pyridine ≤ 200 ppm Ethanol ≤ 10% v/v (excipient)	DMA ≤ 50 ppm (LOQ) Acetonitrile ≤ 100 ppm Pyridine ≤ 50 ppm (LOQ) Ethanol = 6.5 ± 0.5 v/v	DMA ≤ 20 ppm (LOQ) Acetonitrile ≤ 100 ppm Pyridine ≤ 20 ppm (LOQ) Ethanol ≤ 10% v/v

### In-vitro studies

Independently synthesised samples of the structurally related chemical impurities were assessed for their affinity at human GABA_A_R, with the parent FMZ and a control compound, PF-06372865. The affinities obtained for the literature compound PF-06372865 of 0.28, 2.4, 2.8 and 7.2 nM at the α1, α2, α3 and α5-GABA_A_R subtypes, respectively are comparable to previously published values (0.18, 2.9, 1.1 and 18 nM;) (Atack et al. 2006). The affinity of FMZ, OHMZ and HMZ obtained for the α1, α2, α3 and α5-GABAAR subtypes are outlined in Table 2. (Table 2).

**Table 2:** Mean Ki values for FMZ, OHMZ, HMZ and PF-06372865 for GABA_A_ subtypes


Compound	Description	Affinity at human GABA_A_R subtypes, nM			
		α1β3γ2	α2β3γ2	α3β3γ2	α5β3γ2
FMZ	Parent	5.9 ± 0.5 *	5.2 ± 0.6	5.4 ± 0.9	1.7 ± 0.3
OHMZ	Minor impurity	5.4 ± 0.6	7.3 ± 1.5	2.7 ± 0.9	0.5 ± 0.1
HMZ	Minor impurity	24 ± 3	15 ± 2	11 ± 4	1.7 ± 0.2
PF-06372865	Control	0.28 ± 0.03	2.4 ± 0.23	2.8 ± 0.26	7.2 ± 0.69

*Values shown are mean ± SEM of n = 4 separate assays

## Discussion

The major limitations in current methods for the radiochemical synthesis of [^18^F]FMZ are the low-yields associated with nucleophilic substitution methods and the stability of the precursor required for the iodonium chemistry. We were conscious that any new method needed to address not only these points but should also be simple to set-up and use analytical methods readily implemented in radiochemistry quality control laboratories. Our preliminary experiments began with assessing whether our previously reported Cu-mediated method (Preshlock et al. 2016b) would be translatable to the cassette-based synthesizer. A notable feature of this method is the use of potassium oxalate (K_2_C_2_O_4_) for the elution of [^18^F]fluoride from the QMA cartridge. The less basic nature of oxalate in comparison to carbonate was found to be crucial to obtain sufficient RCYs. Pleasingly, when a QMA-carbonate conditioned with potassium oxalate (60 mM, 5 mL) was used in combination with an eluent composed of K_222_/K_2_C_2_O_4_/K_2_CO_3_ (30 mM, 15 mM, 2.2 mM, in 20% H_2_O/CH_3_CN, 800 µL), formation of [^18^F]FMZ was observed in 40 ± 2% radiochemical conversion (RCC) (*n* = 3) n.d.c, demonstrating the compatibility of the method on the AIO automated synthesiser. However, using this oxalate-based eluent resulted in substantial losses through the QMA (66 ± 19% recovery, *n* = 3). Switching to a solution of TBA-HCO_3_ as QMA eluent dramatically improved both the reliability of [^18^F]fluoride release and the overall RCY. Volumes as low as 300 µL were sufficient to achieve > 97% recovery, thus shortening the duration of the subsequent drying. A spot test for TBA-HCO_3_ recently entered the European Pharmacopiea (Ph. Eur. Method 2.4.33 Tetrabutylammonium in Radiopharmaceutical Preparations Cardinale et al. 2017; Tanzey et al 2020), giving an official guideline to verify the residual content of TBA-HCO_3_ in the final product vial, which is not the case for oxalate-based eluents. Gratifyingly, commercially available carbonated QMA could be used without the need for manual conditioning of the cartridge. Using this TBA-HCO_3_ elution system, the effect of reagent loading was investigated. Keeping a precursor/copper-adduct molar ratio of 1:1.4, FMZ-BPin precursor loadings of 18 mg, 12 mg and 8 mg gave RCCs of 63 ± 4%, 65 ± 2% and 25 ± 11% (*n* = 3), respectively. Based on these results, a loading of 12 mg of FMZ-BPin (29 µmol) and 27 mg of (Pyr)_4_Cu(OTf)_2_ (40 µmol), each solubilized in 500 µL of DMA, was selected for further studies. For this synthesis, premixing the dried [^18^F]fluoride with FMZ-BPin did not significantly impact the labelling efficiency with RCCs of 65 ± 2% (*n* = 3) and 64 ± 5% (*n* = 4). The effect of the reaction time showed that after 5 min and 10 min RCCs were 57% and 66% (*n* = 2), respectively; later timepoints showed little-to-no increase in conversion. Similarly, no improvement in rate nor conversion was observed at higher temperature (130 °C). The radiolabelling conditions were therefore set to 120 °C for 12 min. The last part of the optimization was focused on the purification steps. Initial attempts of pre-purification using Plus Light cartridges (SepPak tC18 or Oasis HLB) proved unsuccessful as the radioactive product was not trapped. Increasing the sorbent load and switching to a SepPak Plus Short tC18 cartridge allowed quantitative and selective product trapping, with ≥ 98% of unreacted [^18^F]fluoride being eluted through. Release of crude [^18^F]FMZ was achieved with a 23% aqueous ethanolic solution. Losses on the cartridge were found negligible (1.5 ± 0.7%, *n* = 6). Following elution, the material was purified by semi-preparative HPLC. Isocratic elution with 25% ethanol in 10 mM phosphate buffer (pH 7.2) at 4 mL/min on a Phenomenex Luna® 5 μm C18(2) (250 × 10 mm) proved optimal, allowing isolation of pure [^18^F]FMZ with a retention time of ≈ 15 min and an 8.0 mL collected fraction. The low ethanol content allowed direct isolation of [^18^F]FMZ in the final product vial, without the need for reformulation; a simple dilution to 25 mL with a solution of sodium ascorbate in saline sufficed to reach a final ethanol content compliant with the European Pharmacopoeia requirements.

Pleasingly, the optimized set-up was successfully reproduced at both sites without any alterations or modifications. The n.d.c. RCY of [^18^F]FMZ was 43 ± 2% (*n* = 5) starting from 300–340 MBq of fluorine-18. This compares well with a n.d.c RCY of 35 ± 5% (*n* = 3) was achieved starting from 23.6 ± 5.8 GBq of [^18^F]fluoride, the activity of [^18^F]FMZ in the product vial was between 6.9–9.4 GBq (n.d.c). These RCYs are a significant improvement on the S_N_Ar method (~ 10%) (vide supra), and comparable to the iodonium method by Moon et al. While activity yields have not been widely reported for [^18^F]FMZ, a detailed report by Schirrmacher et al. (2012), reports activity yields of 789 MBq (*n* = 3) starting from 11–16 GBq of [^18^F]fluoride. In comparison, this current method produces 7–9 GBq of [^18^F]FMZ, highlighting the advance using this copper-mediated methods to produce multiple-patients doses from a single production run. Preliminary studies on the stability of the FMZ-BPin precursor indicate it is stable for prolonged storage at room temperature (> 9 months). Quality control on the final product confirmed that it met the predetermined acceptance criteria of radiochemical purity, pH, residual solvent and copper content to meet GMP requirements (Table 1). The molar activity of [^18^F]FMZ was 312 GBq/µmol at the end of synthesis. Copper content was conveniently measured by semi-quantitative test-strips to confirm that copper content was below the established permitted daily exposure (PDE) for copper impurities (300 µg/day), the accuracy of the test-strips was validated by independent ICP-MS analysis (see supporting information). Chemical impurities were identified and quantified by comparison with the retention times of reference standards by HPLC (Mossine et al 2018). To gain initial pharmacological data on OHMZ and HMZ, in-vitro testing was undertaken to determine their affinity for GABA_A_R in comparison to FMZ and a known reference compound, PF-06372865 (Table 2). The compounds HMZ, OHMZ and FMZ were found to have similar Ki values for all of the GABA_A_ subtypes tested, with the exception of HMZ having a slightly higher Ki for α1β3γ2. While OHMZ was formed during the synthesis up to 3.7 ± 0.8 µg/V, this value is lower than the limiting amount of desmethylflumazenil (5 µg/V) and flumazenil (50 µg/V) in the synthesis of [^11^C]FMZ (European Pharmacopeia. 9.0; 1137–1139), and below the proposed micro-dosing limit of 100 µg (Koziorowski et al. 2017). The similarity between the affinity of FMZ and OHMZ towards the GABA_A_ allows us to calculate an effective molar activity of 214 GBq/µmol comparable to typical molar activities of [^11^C]FMZ, which has been used clinically with molar activities as low as 37 GBq/µmol (Pappata et al. 1988).

## Conclusion

We have developed an automated radiosynthesis of [^18^F]flumazenil from the corresponding pinacol-boronate ester precursor. The optimized process benefits from short production time and simplified purification procedures, whilst ensuring clinically useful RCYs and a final product quality compliant for human injection. The results constitute a significant improvement on previously reported methods for preparing [^18^F]flumazenil, and highlight the appeal of aryl pinacol-boronate ester precursors for automated aromatic ^18^F-labelling. The automatization on the AIO platform ensures fast, reliable and high-yielding synthesis of [^18^F]FMZ, making the process suitable for off-site multi-dose production at a low cost. Overall, this process has the potential to revolutionize and expand the use of [^18^F]flumazenil in PET-neuroimaging, and the use of the copper-mediated ^18^F-fluorination of boronate esters for clinical production.

## Supplementary Information

**Additional file 1**. Supplementary information.

## Data Availability

All data generated or analysed during this study are included in this published article and its supplementary information files.

## Abbreviations

AIO: AllinOne
FDG: Fluoro-2-deoxy-D-glucose FMZ: flumazenil
FMZ-BPin: Ethyl 5-methyl-6-oxo-8(4,4,5,5-tetramethyl-1,3,2-dioxaborolan-2-yl)-5,6-dihydro-4H-benzo[f]imidazo[1,5-a][1,4]diazepine-3-carboxylate
GABA: γ-Aminobutyric acid-A
GMP: Good manufacturing practice
HMZ: Ethyl 5-methyl-6-oxo-5,6-dihydro-4H-benzo[f]imidazo[1,5-a][1,4]diazepine-3-carboxylate
HPLC: High-performance liquid chromatography
ICP-MS: Inductively coupled plasma mass spectrometry
n.d.c: Non-decay corrected
OHMZ: Flumazenil impurity B
PDE: Permitted daily exposure
PET: Positron emission tomography
QC: Quality control
RCY: Radiochemical yield
SPE: Solid phase extraction
TBA-HCO_3_: Tetrabutylammonium bicarbonate
TLC: Thin-layer chromatography

## References

[CR1] Atack JR, Wafford KA, Tye SJ (2006). TPA023 [7-(1,1-dimethylethyl)-6-(2-ethyl-2*H*-1,2,4-triazol-3-(2-fluorophenyl)-1,2,4-triazolo[4,3-*b*]pyridazine], an agonist selective for α2- and α3-containing GABA_A_ receptors, is a non-sedating anxiolytic in rodents and primates. J Pharmacol Exp Ther.

[CR2] Atack JR, Wong DF, Fryer TD (2010). Benzodiazepine binding site occupancy by the novel GABA_A_ receptor subtype-selective drug 7-(1,1-dimethylethyl)-6-(2-ethyl-2*H*-1,2,4-triazol-3-ylmethoxy)-3-(2-fluorophenyl)-1,2,4-triazolo[4,3-*b*]pyridazine (TPA023) in rats, primates, and humans. J Pharmacol Exp Ther.

[CR3] Brooks AF, Topczewski JJ, Ichiishi N, Sanford MS, Scott PJH (2014). Late-stage [^18^F]fluorination: new solutions to old problems. Chem Sci.

[CR4] Cardinale J, Martin R, Remde Y (2017). Procedures for the GMP-compliant production and quality control of [^18^F]PSMA-1007: a next generation radiofluorinated tracer for the detection of prostate cancer. Pharmaceuticals.

[CR5] Deng X, Rong J, Wang L (2019). Chemistry for positron emission tomography: recent advances in ^11^C-, ^18^F-, ^13^N-, and ^15^O-labeling reactions. Angew Chem Int Ed.

[CR6] Eng W, Atack JR, Sanabria S, et al, Occupancy of human brain GABA_A_ receptors by the α5 subtype-selective benzodiazepine site inverse agonist α5IA as measured using [^11^C]flumazenil PET imaging. Neuropharmacology. 2010;59635–639 10.1016/j.neuropharm.2010.07.024 20696179

[CR7] Flumazenil (*N*-[^11^C]methyl injection 01/2008:1917, Radiopharmaceutical preparations. In European Pharmacopeia. 9.0; 1137–1139.

[CR8] Flumazenil, European Pharmacopeia, 10.7; 2655

[CR9] Gu Z-Q, Wong G, Dominguez S (1993). Synthesis and evaluation of imidazo[1,5-*a*][1,4]benzodiazepine esters with high affinities and selectivities at “diazepam-insensitive” benzodiazepine receptors. J Med Chem.

[CR10] Guibbal F, Isenegger PG, Wilson TC (2020). Manual and automated Cu-mediated radiosynthesis of the PARP inhibitor [^18^F]olaparib. Nat Protoc.

[CR11] Heiss W-D, Kracht L, Grond M (2000). Early [^11^C]flumazenil/H_2_O positron emission tomography predicts irreversible ischemic cortical damage in stroke patients receiving acute thrombolytic therapy. Stroke.

[CR12] Holthoff VA, Koeppe RA, Frey KA (1993). Positron emission tomography measures of benzodiazepine receptors in Huntington’s disease. Ann Neurol.

[CR13] Koepp MJ, Labbé C, Richardson MP (1997). et aal, Regional hippocampal [^11^C]flumazenil PET in temporal lobe epilepsy with unilateral and bilateral hippocampal sclerosis. Brain.

[CR14] Koziorowski J, Behe M, Decristoforo C (2017). Position paper on requirements for toxicological studies in the specific case of radiopharmaceuticals. EJNMMI Radiopharm Chem.

[CR15] Kumar P, Nagaraj C, Joshi R (2021). Radiosynthesis of [^18^F]flumazenil for imaging benzodiazepine receptors and its evaluation in human volunteers using simultaneous PET-MRI. J Radioanal Nucl Chem.

[CR16] Malizia AL, Cunningham VJ, Bell CJ, Liddle PF, Jones T, Nutt DJ (1998). Decreased brain GABA_A_-benzodiazepine receptor binding in panic disorder: preliminary results from a quantitative PET study. Arch Gen Psychiatry.

[CR17] Massaweh G, Schirrmacher E (2009). la Fougere, et al, Improved work-up procedure for the production of [^18^F]flumazenil and first results of its use with a high-resolution research tomograph in human stroke. Nucl Med Biol.

[CR18] Moon BS, Kil HS, Park JH (2011). Facile aromatic fluorination of [^18^F]flumazenil from diaryliodonium salts with evaluation of their stability and selectivity. Org Biomol Chem.

[CR19] Moon BS, Park JH, Lee HJ, Lee BC, Kim SE (2014). Routine production of [18F]flumazenil from iodonium tosylate using a sample pretreatment method: a 2.5 year production report. Mol Imaging Biol.

[CR20] Mossine AV, Brooks AF, Ichiishi N (2017). Development of customized [^18^F]fluoride elution techniques for the enhancement of copper-mediated late-stage radiofluorination. Sci Rep.

[CR21] Mossine AV, Brooks AF, Bernard-Gauthier V (2018). Automated synthesis of PET radiotracers by copper mediated-mediated ^18^F-fluorination of organoborons: importance of the order of addition and competing protodeborylation. J Labelled Comp Radiopharm.

[CR22] Mossine AV, Tanzey SS, Brooks AF (2020). Synthesis of high-molar-activity [^18^F]6-fluoro- ʟ-DOPA suitable for human use via Cu-mediated fluorination of a BPin precursor. Nat Protoc.

[CR23] Nickolls SA, Gurrell R, van Amerongen G (2018). Pharmacology in translation: the preclinical and early clinical profile of the novel α2/3 functionally selective GABA_A_ receptor positive allosteric modulator PF-06372865. Br J Pharmacol.

[CR24] Pappata S, Samson Y, Chavoix C (1988). Regional specific binding of [^11^C]RO151788 to central type benzodiazepine receptors in human brain: quantitative evaluation by PET. J Cereb Blood Flow Metab.

[CR25] Ph. Eur. Method 2.4.33 Tetrabutylammonium in Radiopharmaceutical Preparations

[CR26] Preshlock S, Tredwell M, Gouverneur V (2016). ^18^F-Labelling of arenes and heteroarenes for applications in positron emission tomography. Chem Rev.

[CR27] Preshlock S, Calderwood S, Verhoog S (2016). Enhanced copper-mediated ^18^F-fluorination of aryl boronic esters provides eight radiotracers for PET applications. Chem Commun.

[CR28] Ryvlin P, Bouvard S, Le Bars D (2020). Clinical utility of flumazenil-PET versus [^18^F]fluorodeoxyglucose-PET and MRI in refractory partial epilepsy: a prospective study in 100 patients. Exp Neurol.

[CR29] Ryzhikov NN, Seneca N, Krasikova RN (2005). Preparation of highly specific radioactivity [^18^F]flumazenil and its evaluation in cynomolgus monkey by positron emission tomography. Nucl Med Biol.

[CR30] Savic I, Persson A, Roland P, Pauli S, Sedvall G, Widén L (1988). In-vivo demonstration of reduced benzodiazepine receptor binding in human epileptic foci. Lancet.

[CR31] Schirrmacher R, Massaweh G, Kovacevic M, Wängler C, Thiel A, Scott PJH, Hockley BG (2012). Synthesis of [^18^F]flumazenil ([^18^F]FMZ). Radiochemical syntheses: radiopharmaceuticals for positron emission tomography.

[CR32] Tanzey SS, Mossine AV, Sowa AR (2020). A spot test for determination of residual TBA levels in ^18^F-radiotracers for human use using Dragendorff reagent. Anal Methods.

[CR33] Tredwell M, Preshlock S, Taylor NJ (2014). A general copper-mediated nucleophilic ^18^F fluorination of arenes. Angew Chem Int Ed.

[CR34] Vaulina D, Nasirzadeh M, Gomzina N (2018). Automated radiosynthesis and purification of [^18^F]flumazenil with solid phase extraction. Appl Radiat Isot.

[CR35] Vivash L, Gregoire M-C (2013). Lau Ew, et al, ^18^F-Flumazenil: a γ-aminobutyric acid A-specific PET radiotracer for the localization of drug-resistant temporal lobe epilepsy. J Nucl Med.

[CR36] Wright JS, Kaur T, Preshlock S (2020). Copper-mediated late-stage radiofluorination: five years of impact on preclinical and clinical PET imaging. Clin Transl Imaging.

[CR37] Zlatopolskiy BD, Zischler J, Krapf P (2015). Copper-mediated aromatic radiofluorination revisited: efficient production of PET tracers on a preparative scale. Chem Eur J.

