# Supplementary material for: Multi-patient dose synthesis of [18F]Flumazenil via a copper-mediated 18F-fluorination
Source: EJNMMI Radiopharm Chem. 2022 Mar 20;7:5. doi: 10.1186/s41181-022-00158-z (PMC8934836; doi:10.1186/s41181-022-00158-z)
Supplement: Supplementary file 1 — Additional file 1. Supplementary information. [file 41181_2022_158_MOESM1_ESM.pdf]

# Supplementary Information

## Multi-patient dose synthesis of [$^{18}$F]Flumazenil via a copper-mediated 18F-fluorination

Thibault Gendron,[1]* Gianluca Destro,[2]* Natan J. W. Straathof,[2] Jeroen B. I. Sap,[2] Florian Guibbal,[2] Charles Vriamont,[1] Claire Caygill,[3] John R. Atack,[3] Andrew J. Watkins,[4] Christopher Marshall,[4] Rebekka Hueting,[4] Corentin Warnier,[1] Véronique Gouverneur,[2] Matthew Tredwell[4,5]

1) Trasis, Rue Gilles Magnée, 90 – 4430 Ans, Belgium; 2) Chemistry Research Laboratory, University of Oxford, 12 Mansfield Road, Oxford, OX1 3TA, UK; 3) Medicines Discovery Institute, Cardiff University, Main Building, Park Place, Cardiff CF10 3AT; 4) Wales Research and Diagnostic PET Imaging Centre, Cardiff University, University Hospital of Wales, Heath Park, Cardiff, CF14 4XN, UK; 5) School of Chemistry, Cardiff University, Main Building, Cardiff, CF10 3AT, UK

* These authors contributed equally to the study

Emails for correspondence: tredwellm@cardiff.ac.uk; veronique.gouverneur@chem.ox.ac.uk

### Table of Contents

**1. Automated radiosynthesis of [$^{18}$F]flumazenil** **2**
1.1 General considerations **2**
1.2 Cassette assembly **2**
1.3 Reagent kit **3**
1.4 Process description **4**
1.5 Quality control **5**
**2. In-vitro studies** **13**
**3. Semi-preprarative HPLC traces** **14**
**4. References** **14**

# 1. Automated Radiosynthesis of [$^{18}$F]Flumazenil

## 1.1. General Considerations

The radiosynthesis of [$^{18}$F]flumazenil ([$^{18}$F]FMZ) was carried-out on an AllinOne synthesizer (Trasis, Belgium), equipped with 18 valves and an integrated HPLC+UV purification system. Fluid pathways, consumables, and tetrabutylammonium bicarbonate solution were supplied by Trasis. Solid phases extraction (SPE) cartridges were purchased from Waters. The air-intake drying cartridge was manually assembled by filling an empty medium-sized cartridge (BGB Analytik Vertrieb GmbH) with molecular sieves powder, 4 Å, 325 mesh particle size (Sigma-Aldrich). Sterile 0.9% saline solution bottles were obtained from Baxter. Semi-preparative HPLC purification of [$^{18}$F]FMZ was achieved on a Luna® 5 µm C18(2) 100 Å column (10 x 250 mm) (Phenomenex, Part#: 00G-4252-N0). Radiochemical yields were calculated from the ratio of the activity measured in the final product vial at end of synthesis and the initial activity in the [$^{18}$F]fluoride in $^{18}$O-water solution from the cyclotron, or radioactivity delivered from the cyclotron; these are given non-decay corrected.

## 1.2. Cassette Assembly

The cassette was assembled using standard Trasis consumables as follows (Figure S1 and Table S1).

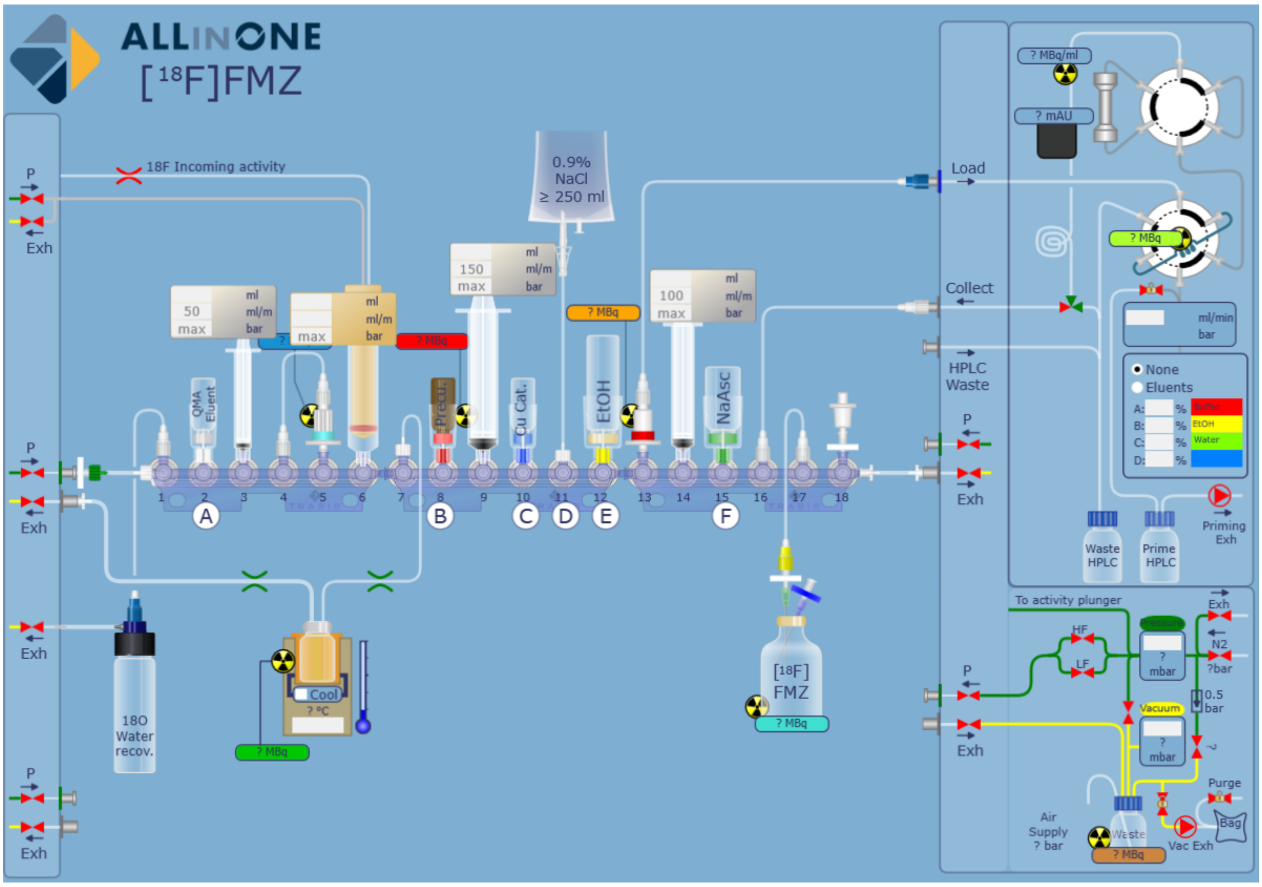


**Figure S1**. Layout of the cassette/kit used for automated radiosynthesis of [$^{18}$F]FMZ on the AllinOne.

**Table S1.** List of cartridges required to assemble the cassette.

| Position | Item | Qty | Unit | Trasis order# |
|---|---|---|---|---|
| 5 | Sep-Pak Accell Plus - QMA Carbonate Plus Light Cartridge - 46 mg | 1 | pc | 6917 |
| 13 | Sep-Pak tC18 Plus Short Cartridge - 400 mg sorbent | 1 | pc | 3057 |
| 18 | Air-intake drying cartridge (MS 4Å, 325 mesh) | 1 | pc | - |

## 1.3. Reagent Kit

**Table S2.** List of chemicals in the reagent kit required for one [$^{18}$F]FMZ production.

| Position | Item | Composition | Container | Colour | Storage |
|---|---|---|---|---|---|
| 2 | QMA Eluent | Solution of $TBA\text{-}HCO_3$ in acetonitrile/water (450 µL). | 4 mL clear glass vial | Silver | Freezer |
| 8 | Precursor | FMZ-BPin precursor (12 mg, 30 µmol) | 4 mL amber glass vial | Red | Freezer |
| 10 | Copper catalyst | Tetrakis(pyridine)copper(II) triflate (27 mg, 40 µmol) | 4 mL amber glass vial | Blue | Freezer |
| 11 | Bag of saline | 0.9% Saline for injection (250 mL) | Plastic bag | - | RT |
| 12 | Ethanol | Ethanol absolute EMSURE Eu. Ph. (10 mL) | 20 mL clear glass vial | Yellow | Fridge |
| 15 | Sodium ascorbate | Sodium Ascorbate Eu. Ph. (125 mg) | 10 mL clear glass vial | Green | Fridge |
| - | Solvent for precursor and copper catalyst | *N,N*-Dimethylacetamide anhydrous, Zer$O_2$®, 99.8% (2 mL) | 2 mL glass ampule | - | Fridge |
| HPLC Solvent A | Aqueous buffer | Phosphate buffer 10 mM in water for injection (250 mL) | - | Red | RT |
| HPLC Solvent B | Ethanol | Ethanol absolute EMSURE Eu. Ph. (250 mL) | - | Yellow | RT |
| HPLC Solvent C | Water | Water for injection (500 mL) | - | Green | RT |

### 1.4. Process Description

#### 1.4.1. Preliminary steps

Both the precursor and the tetrakis(pyridine)copper(II) triflate are solubilized manually before starting the procedure; this is to ensure solubilisation of the copper reagent which has a limited solubility in *N*,*N*-dimethylacetamide. The standard procedure to prepare these vials is given below:

1) Break-open the anhydrous *N*,*N*-dimethylacetamide ampule
2) Using a 1 mL syringe equipped with a 23G needle (Sterican, L 80 mm), withdraw 0.60 mL of anhydrous *N*,*N*-dimethylacetamide
3) Dispense 0.50 mL of the solvent into the precursor vial (amber glass, red crimp), piercing through the centre of the septum
4) Using a new 1 mL syringe equipped with a 23G needle (Sterican, L 80 mm), withdraw 0.60 mL of anhydrous *N*,*N*-dimethylacetamide
5) Dispense 0.50 mL of the solvent into the copper vial (clear glass, blue crimp), piercing through the centre of the septum
6) Shake both vials vigorously; the copper reagent requires up-to 1 min vortexing at maximum speed to fully solubilize
7) Check visually for remaining solid in the vials before placing on the cassette. If crystals are still visible, repeat step 6.

#### 1.4.2. Cassette and reagent placements

When prompted by the command software, the cassette is placed on the machine. After a complete self-check of the equipment and the cassette, the reagents are placed on the cassette as shown on Figure S1.

#### 1.4.3. Radiosynthesis

The radiosynthesis process is fully automated, including cartridges conditioning, HPLC conditioning, synthesis and HPLC purification, followed by product transfer to the final vial. A brief overview of the synthesis program is given hereafter.

1) The [$^{18}$F]fluoride in $^{18}$O-water is received from the cyclotron and transferred into the activity reservoir on V6. In parallel, the HPLC pump is started to equilibrate the column in 25% EtOH in buffer at a flowrate of 3.0 mL/min.
2) The radioactive solution is subsequently transferred through the QMA cartridge, effectively trapping the [$^{18}$F]fluoride, and directed toward the enriched water collection vial (V6-5-4-1).
3) After a brief flush of the QMA and lines, the QMA eluent is withdrawn from its vial (V2) using the 3 mL BD syringe (V3) and is subsequently passed through the QMA toward the reactor to release the activity (V4-5-7).
4) The [$^{18}$F]fluoride is then azeotropically dried under vacuum/nitrogen flow using a gradient of temperature (80-125 °C).
5) The reactor is then actively cooled to 75 °C using a flow a compressed air.
6) The precursor and the tetrakis(pyridine)copper(II) triflate in *N*,*N*-dimethylacetamide are successively added into the reactor by pressure-transfer.
7) 20 mL of dry air are withdrawn from V18 using the 20 mL BD Syringe and then added into the reactor in such a way that the bubbling remains gentle, limiting losses on the wall and cap of the reactor.
8) The reactor temperature is set to 120 °C while a second flush of dry air is added into the reactor (20 mL, from V18 using the 20 mL BD Syringe). At the end of the addition, the reactor is closed, and a reaction timer is set to 12 min.
9) In parallel to the reaction, the tC18 cartridge (V13) is conditioned with 5 mL EtOH (V12) and 10 mL saline (V11), using the 20 mL BD Syringe.

10) The quench solution is then prepared by withdrawing 3 mL and 3.5 mL of saline (V11) in the 3 mL and 20 mL BD syringes, respectively.
11) At the end of the 12 min reaction, the reactor is actively cooled to 50 °C before the reaction mixture is diluted with saline from the 20 mL BD Syringe (V9-7).
12) The crude reaction mixture is withdrawn into the 20 mL BD Syringe.
13) The reactor is subsequently rinsed with 2 × 3 mL of saline using the 3 mL BD Syringe, each rinse being withdrawn into the 3 mL BD Syringe.
14) The content of the 20 mL BD Syringe is diluted to 22 mL with saline (V11-9) before being passed through the SepPak tC18 plus short cartridge (V13). The eluate is directed toward the HPLC waste bottle.
15) The cartridge is subsequently washed with 10 mL of saline (V11) using the 20 mL BD Syringe. The eluate is directed toward the HPLC waste bottle.
16) Crude [$^{18}$F]FMZ is released from the tC18 cartridge by injecting 12 mL of a 23% ethanolic solution prepared in the 20 mL BD Syringe from the ethanol vial (V12) and the saline bottle (V11). The eluate is directed toward the HPLC injection loop.
17) As the injection loop is turned into the Inject position, the acquisition software starts monitoring the UV, the radioactivity, the flowrate, and the pressure in the HPLC system. HPLC conditions 25 % EtOH in 10 mM phosphate buffer (pH 7.2) on a Luna 5 µm C18 column (100 Å, 10 x 250 mm)..
18) Whilst the semi-preparative purification proceeds, the sodium ascorbate (V15) is solubilized with 4 mL of saline (V11) using the 10 mL BD Syringe. The resulting solution is subsequently transferred to the final product vial (FPV) (V17).
19) After approximately 18 min, the [$^{18}$F]FMZ elutes from the column and is collected by switching the collection valve as well as V16-17 to direct the flow toward the FPV.
20) The collection is manually stopped or stopped automatically after 2.25 min, whichever happens first.
21) The isolated [$^{18}$F]FMZ is further diluted with saline using the 10 mL BD Syringe to reach a final volume of 25 mL.
22) After flushing the delivery line with nitrogen, the final product is ready for dispensing and quality control.

#### 1.4.4. End of production

All reagent vials are emptied, and the cassette is thoroughly washed to reduce the residual radiation to the lowest level achievable. In parallel, the HPLC column is washed successively with water for injection (to remove phosphate salts from the system) and pure ethanol.

### 1.5. Quality Control

#### 1.5.1. Final Volume

The total volume of solution in the final product vial was determined either by weighing the FPV (knowing the initial tare of the vial) or by measuring the activity concentration.

#### 1.5.2. Appearance

The content of the final product vial was visually assessed behind appropriate shielding. The solution must be clear, colourless, and free of visible particulate matter.
*Results*: all final product solutions were clear, colourless, and free of visible particulate matter.

#### 1.5.3. pH

pH was measured using a Mettler-Toledo Five-Easy pH meter or Mettler-Toledo SevenMulti pH meter, calibrated at pH 4.0, 7.0 and 10.0 with correction for the temperature. The results were cross-checked with two pH paper strips of different ranges.

### 1.5.4. Tracer Identity

The identity of the radiochemical product was confirmed by coelution with the non-radioactive analogue [$^{19}$F]flumazenil authentic standard (Enzo Life Sciences or Tokyo Chemical Industry (TCI) UK) (Figure S2a (Trasis), S2b (Cardiff)).

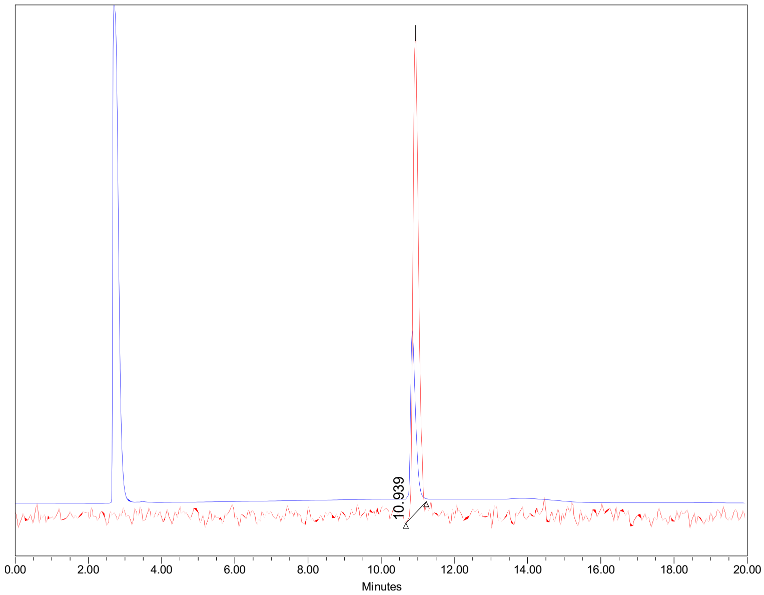


Column: XBridge shield RP18 5 µm (4.6x250 mm)
Flowrate: 1.000 mL/min
Injection: 3 µL

*Gradient*

| Time (min) | $H_2O$ + 0.1% TFA (%) | MeCN + 0.1% TFA (%) |
|---|---|---|
| 0 | 95 | 5 |
| 10 | 50 | 50 |
| 12 | 10 | 90 |
| 15 | 10 | 90 |
| 20 | 95 | 5 |

**Figure S2a** Red: radioactivity detector; blue UV@246 nm. The highest UV peak at approx. 3 min is due to the sodium ascorbate in the formulation.

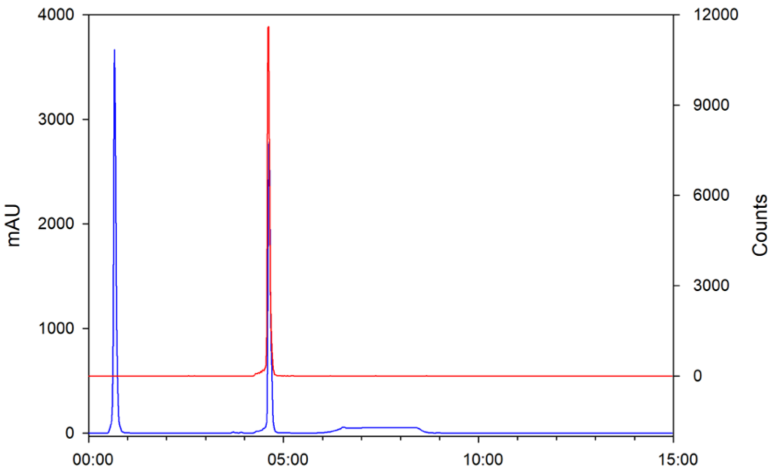


Column: Chromolith® Performance RP-18 (100 × 4.6 mm)
Flowrate: 3.000 mL/min
Injection: 10 µL

*Gradient*

| Time (min) | $H_2O$ + 0.1% TFA (%) | MeCN + 0.1% TFA (%) |
|---|---|---|
| 0 | 95 | 5 |
| 5 | 70 | 30 |
| 5.5 | 10 | 90 |
| 7.5 | 10 | 90 |
| 8 | 95 | 5 |
| 10 | 95 | 5 |
| 15 | 95 | 5 |

**Figure S2b**. Red: radioactivity detector; blue UV@246 nm Coelution of the isolated fraction with the [$^{19}$F]FMZ standard (HPLC).

### 1.5.5. Radiochemical Purity

The radiochemical purity was primarily assessed by radio-TLC (Figure S3a (Trasis), S3b (Cardiff)) and confirmed by radio-HPLC (Figure S4a (Trasis), S4b (Cardiff)).

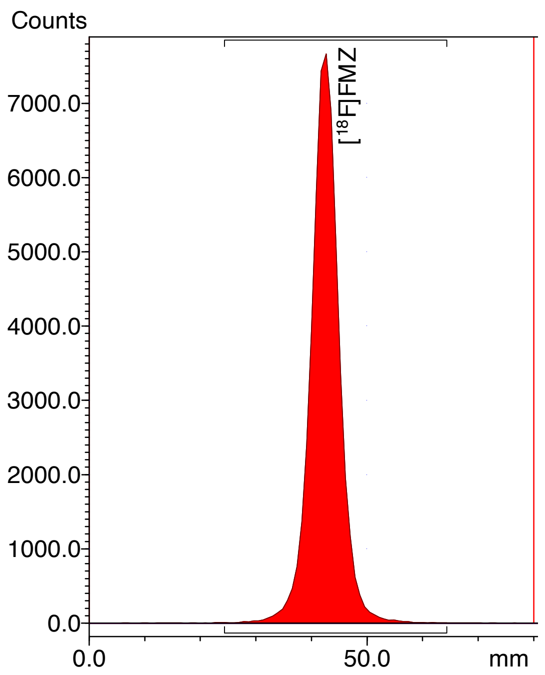


Solid support: Silica gel 60 F254
Eluent: 5% methanol in dichloromethane
Rf = 0.50-0.60

Solid support: Silica gel 60 F254
Eluent: 100% ethyl acetate
Rf = 0.50-0.60

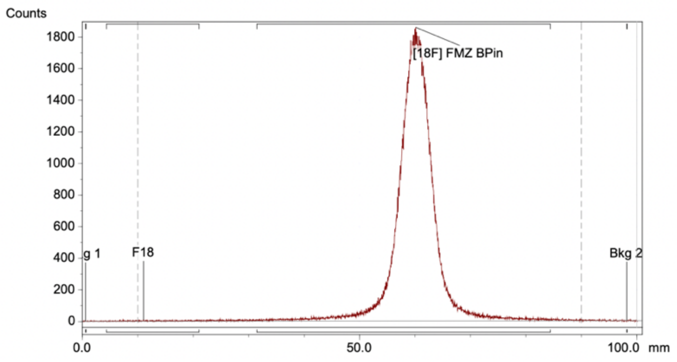


Regions: $^{18}F$

| Name | Start (mm) | End (mm) | Retention (RF) | Area (Counts) | %ROI (%) | %Total (%) | Signal to Noise Ratio | FWHM (mm) |
|---|---|---|---|---|---|---|---|---|
| Bkg 1 | 0.6 | 0.6 | -0.118 | | | | | |
| F18 | 4.4 | 21.0 | 0.013 | 941 | 0.30 | 0.30 | N/A | 16.3 |
| [18F] FMZ BPin | 31.5 | 84.4 | 0.627 | 309296 | 99.70 | 98.98 | N/A | 5.8 |
| Bkg 2 | 98.2 | 98.2 | 1.103 | | | | | |
| 2 Peaks | | | | 310237 | 100.00 | 99.28 | | |

**Figure S3**. Typical radio-TLC of the formulated final product [$^{18}$F]FMZ. If any [$^{18}$F]fluoride residuals were present, a peak would be visible in the 0-10 mm region; not observed here.

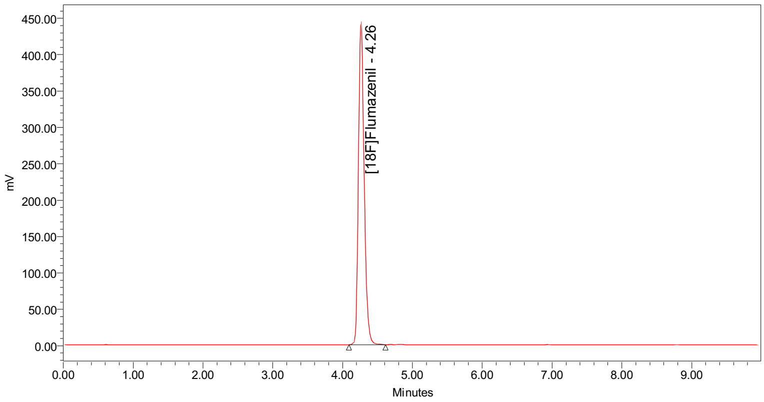


Column: Chromolith® Performance RP-18 (100 × 4.6 mm)
Flowrate: 3.000 mL/min
Injection: 10 µL

*Gradient*

| Time (min) | $H_2O$ + 0.1% TFA (%) | MeCN + 0.1% TFA (%) |
|---|---|---|
| 0 | 95 | 5 |
| 5 | 70 | 30 |
| 5.5 | 10 | 90 |
| 7.5 | 10 | 90 |
| 8 | 95 | 5 |
| 10 | 95 | 5 |

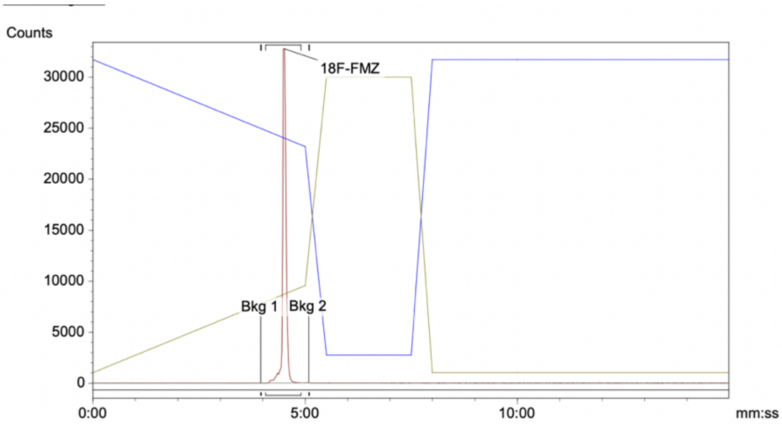


Column: Chromolith® Performance RP-18 (100 × 4.6 mm)
Flowrate: 3.000 mL/min
Injection: 10 µL

*Gradient*

| Time (min) | $H_2O$ + 0.1% TFA (%) | MeCN + 0.1% TFA (%) |
|---|---|---|
| 0 | 95 | 5 |
| 5 | 70 | 30 |
| 5.5 | 10 | 90 |
| 7.5 | 10 | 90 |
| 8 | 95 | 5 |
| 10 | 95 | 5 |
| 15 | 95 | 5 |

**Figure S4**. Typical radio-HPLCs of the formulated final product [$^{18}$F]FMZ.

### 1.5.6. Chemical Purity

The chemical purity was assessed by quantitative HPLC (Figure S5a (Trasis), Figure S5b (Cardiff)). The two main non-radioactive impurities identified in the final formulation were:

- Defluoro 8-hydroxy flumazenil (otherwise known as *USP Flumazenil impurity B*, here abbreviated FMZ-OH)
- [$^{19}$F]Flumazenil

Calibration curves for both compounds were obtained using commercially available authentic standards.

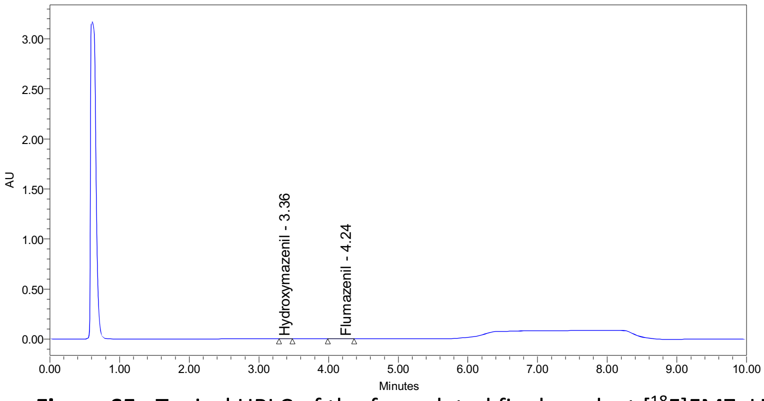


Column: Chromolith® Performance RP-18 (100 × 4.6 mm)
Flowrate: 3.000 mL/min
Injection: 10 µL

*Gradient*

| Time (min) | $H_2O$ + 0.1% TFA (%) | MeCN + 0.1% TFA (%) |
|---|---|---|
| 0 | 95 | 5 |
| 5 | 70 | 30 |
| 5.5 | 10 | 90 |
| 7.5 | 10 | 90 |
| 8 | 95 | 5 |
| 10 | 95 | 5 |

**Figure S5a** Typical HPLC of the formulated final product [$^{18}$F]FMZ; UV detection at 246 nm. The highest UV peak at approx. 0.6 min is due to the sodium ascorbate in the formulation

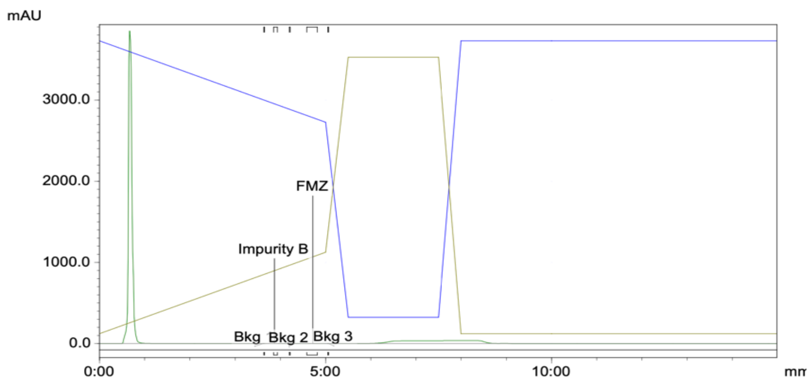


Column: Chromolith® Performance RP-18 (100 × 4.6 mm)
Flowrate: 3.000 mL/min
Injection: 10 µL

*Gradient*

| Time (min) | $H_2O$ + 0.1% TFA (%) | MeCN + 0.1% TFA (%) |
|---|---|---|
| 0 | 95 | 5 |
| 5 | 70 | 30 |
| 5.5 | 10 | 90 |
| 7.5 | 10 | 90 |
| 8 | 95 | 5 |
| 10 | 95 | 5 |
| 15 | 95 | 5 |

**Figure S5b**. Typical HPLC of the formulated final product [$^{18}$F]FMZ; UV detection at 246 nm. The highest UV peak at approx. 0.6 min is due to the sodium ascorbate in the formulation.

Semi-quantitative assessment of *tert*-butyl ammonium (TBA) residuals was carried-out using an in-house purpose-developed spot test (Trasis)[1] (Figure S6a) or a published TLC method using iodine staining (Cardiff) (Figure S6b).[2]

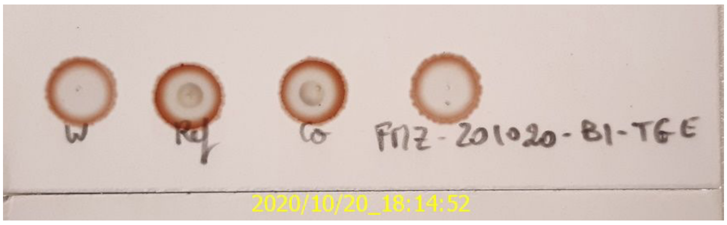


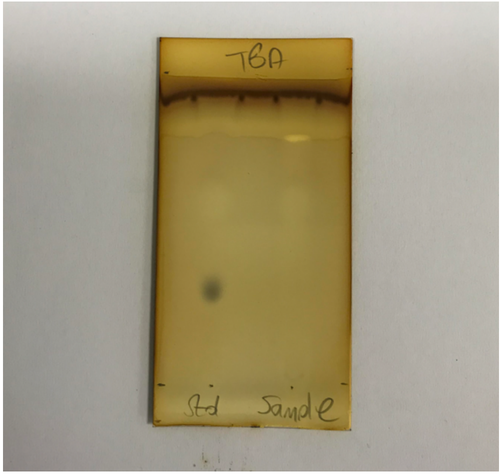


**S6a)** **S6b)**

**Figure S6**. a) Picture of a typical TBA residuals analysis of the formulated final product [$^{18}$F]FMZ using the spot test developed at Trasis. From left to right: W: water (blank); Ref: stock solution of *tert*-butyl ammonium (0.12 mg/mL as $TBA^+$); Co: co-spot consisting of the reference + the sample; Sample. Spotted volume: 2 µL. The presence of TBA+ is revealed by the appearance of a dark blue/green ring at the center of the spot, as visible on spots 2 and 3.
b) Picture of typical TLC of the formulated [$^{18}$F]FMZ using the reported TLC (eluent methanol: ammonium hydroxide 90:10), after 29 minutes the plate is exposed to iodine vapor for 1 minute. From left to right: Std: standard 10 µL spotted volume (concentration 0.22mg/ml). Sample: reformulated [$^{18}$F]FMZ, 10 µL spotted volume

### 1.5.7. Residual Solvents

Quantification of residual solvents was achieved with quantitative GC.

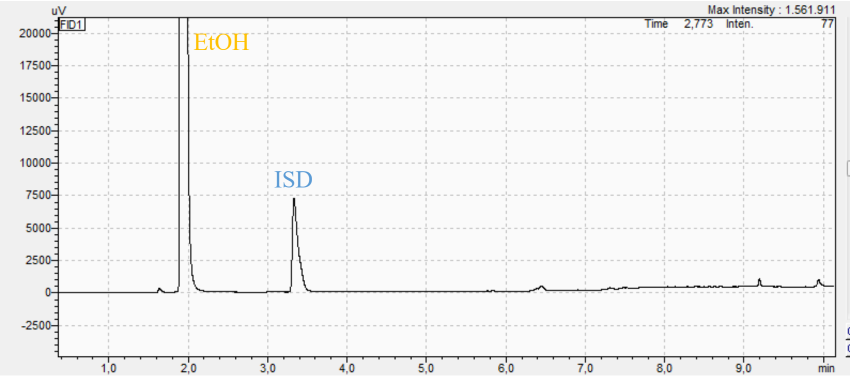


Column: Stabilwax 30 m x 0.25 mm x 0.25 µm
Injected volume: 0.2 µL
Inlet temperature: 280 °C
Column flow: 4 mL/min
Split ratio: 1:25
Purge flow : 10 mL/min
Oven temperature program:
Hold 40 °C for 3.5 min
40 °C/min until 200 °C
Hold 200 °C for 3 min
Detector: FID
$H_2$ flow: 45 mL/min
MakeUp gas: 30 mL/min
Air flow: 450 mL/min
Temperature FID: 300 °C
Rt's: EtOH = 1.92 min; MeCN = 2.76 min; Pyridine = 5 min; DMAc = 6.2 min

**Figure S7a.** GC chromatogram using 1-propanol as internal standard

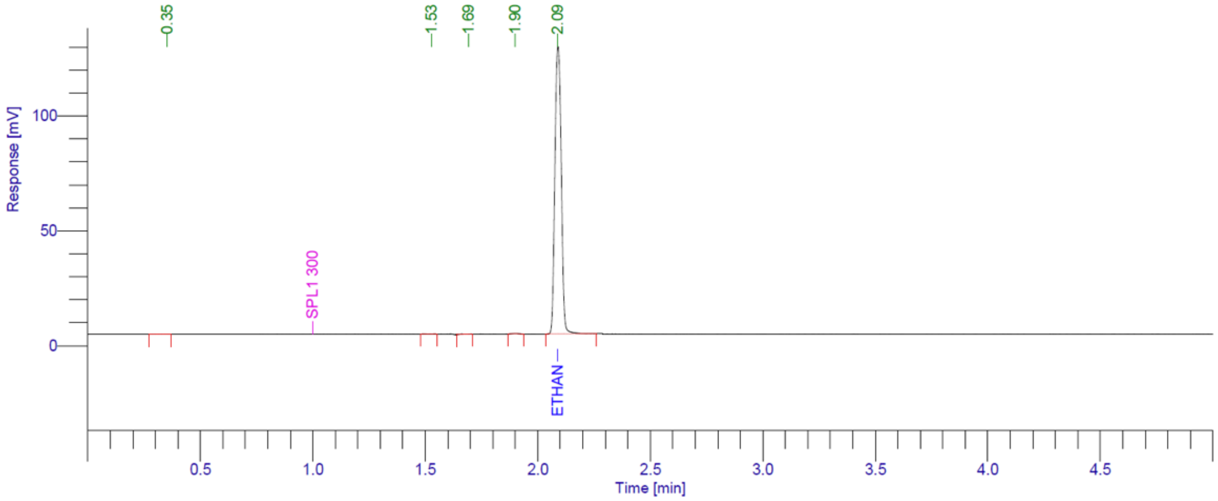


**Figure S7b**. Typical GC chromatogram of the formulated final product [$^{18}$F]FMZ.

### 1.5.8. Copper Content

Semi-quantitative assessment of residual copper content was primarily done using test strips specific to Copper(I)/(II). A small volume (typically 20-50 µL) of sample was evenly distributed on the reactive pad of the test strip; after 30 second, the colour was compared against the reference scale provided by the supplier (Figure S8, top strip). As control, a spiked-sample was prepared by adding 5 µL of a copper(II) stock solution (10.7 mmol/L) to 95 µL of the formulated final product; this yielded a solution at the ICH(Q3) limit concentration of 340 µg/V of copper (Figure S8, bottom strip).

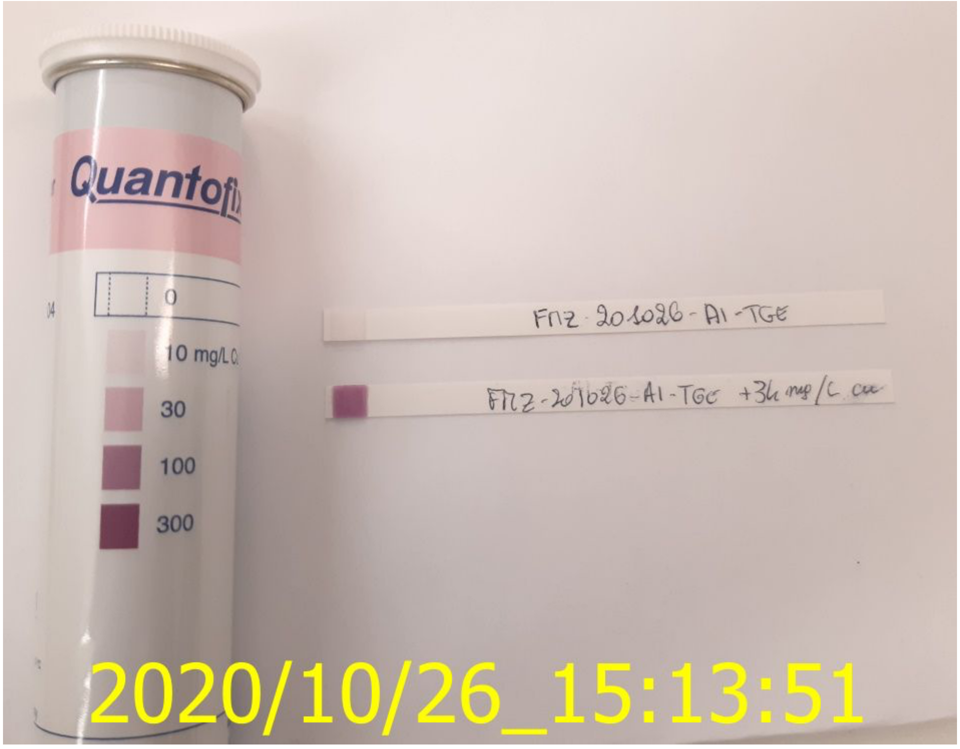


**Figure S8**. Photography of the test used to semi-quantitatively determine residual copper content using Quantofix test strips. Top strip: formulated final product [$^{18}$F]FMZ; Bottom strip: mixture formulated final product [$^{18}$F]FMZ + stock solution of copper(II) to reach a final [$Cu^{I/II}$] = 340 µg/V, where *V* is the maximal injected volume, set to 10 mL.

In addition, three samples were analysed with ICP to determine the residual copper content. Briefly, the samples were prepared as follows: 700 µL of sample were evaporated to dryness in acid cleaned beakers/PFA plastic beakers (to ensure any Cu contamination was kept to a minimum). The dry residue was cooled to room temperature and then carefully 200 µL of 16 M $HNO_3$ were added. The solution was left to rest for two minutes and then 9.8 mL of deionised water were added. Then it was transferred into a 15 mL centrifuge tube. The solution must be clear without the presence of precipitates or insoluble materials. The resulting solution was analysed by ICP (Table S3).

**Table S3.** ICP determination of copper residual solvent in the formulated final product [$^{18}$F]FMZ and comparison with the corresponding test strips.

| Sample | ICP value ng/g | Test strip |
|---|---|---|
| 200703_A1 | 3.051 | - |
| 200708_A1 | 8.504 | 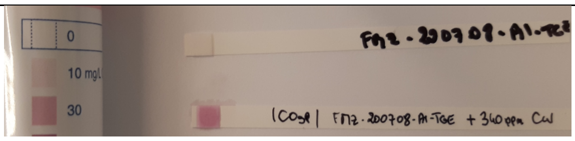  |
| 200709_A1 | 6.890 | 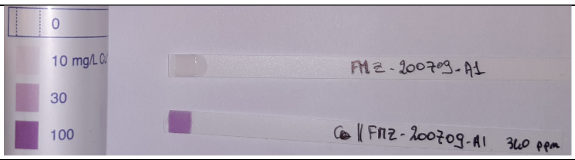  |
| Blank | 3.295 | - |

### 1.5.9 Molar activity

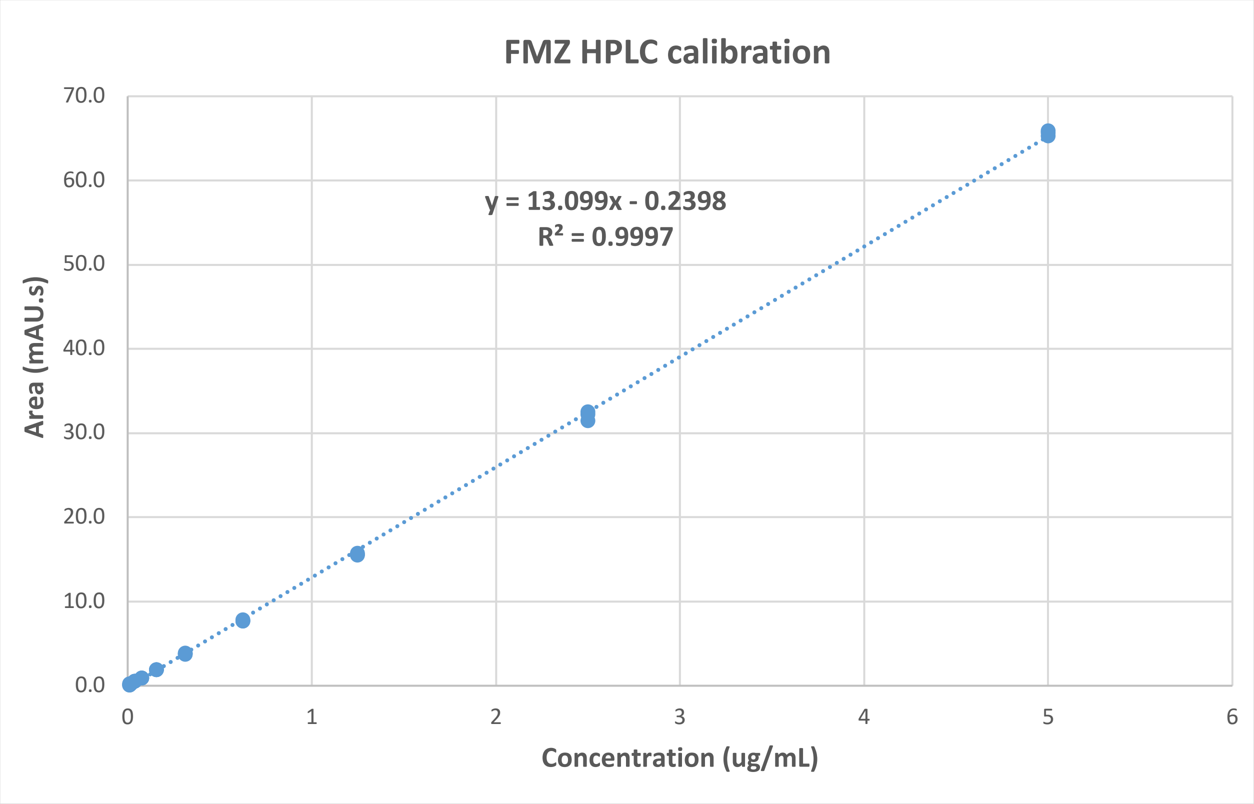


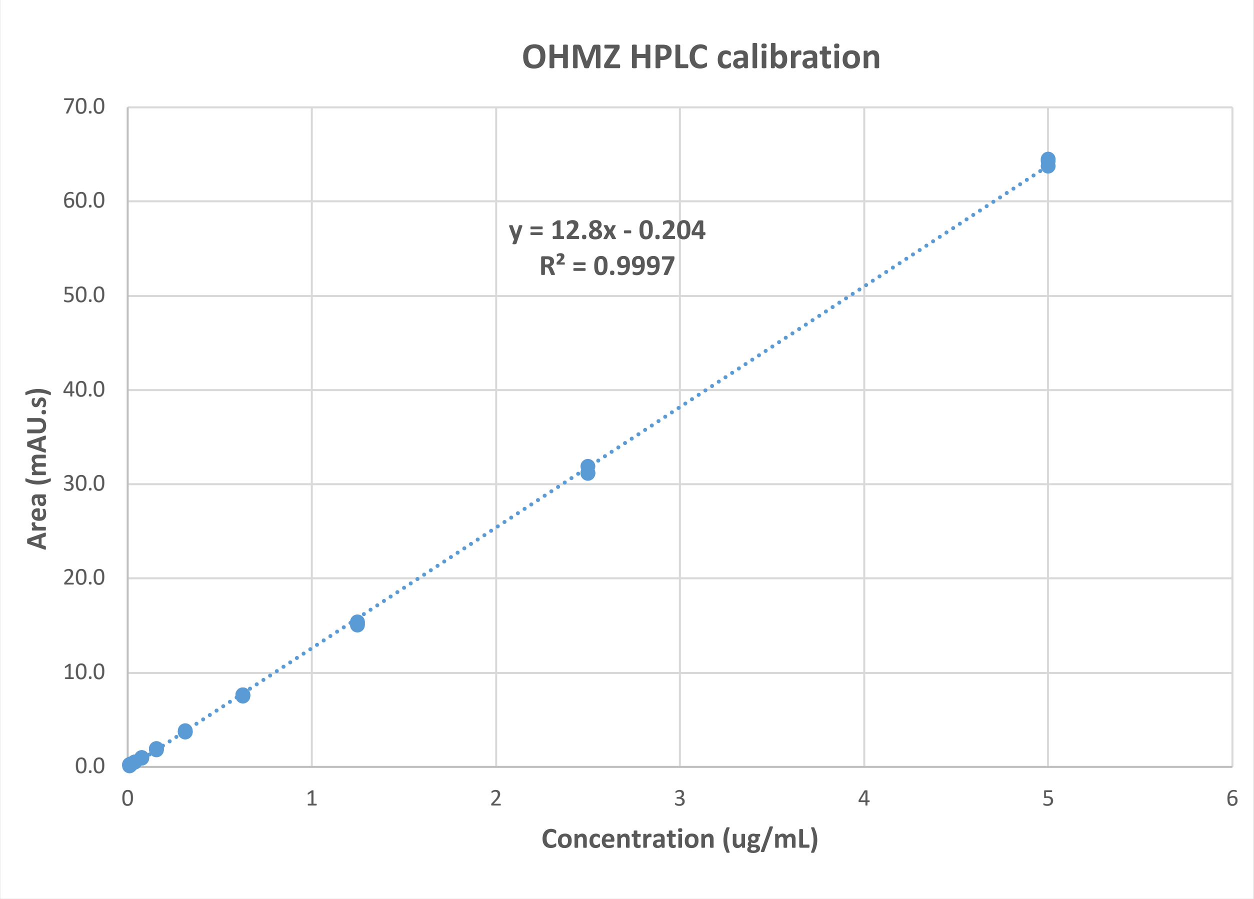

Chromatogram: UV

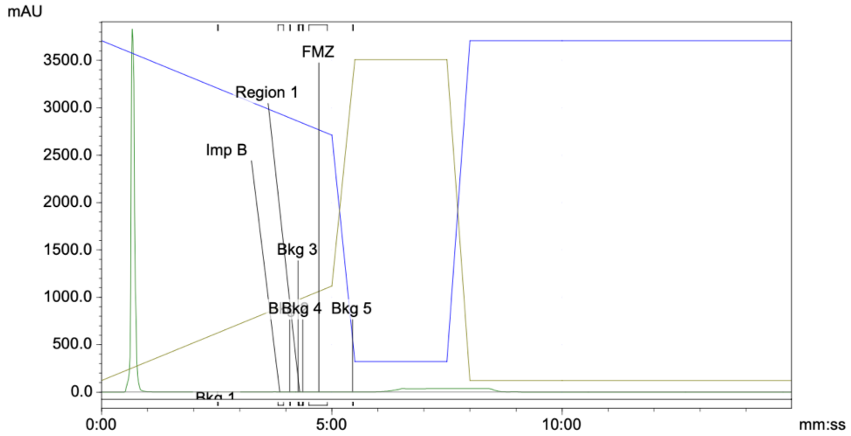


Regions: UV

| Name | Start (mm:ss) | End (mm:ss) | Retention (mm:ss) | Area (mAU·s) | %ROI (%) | %Total (%) | Signal to Noise Ratio | FWHM (mm:ss) |
|---|---|---|---|---|---|---|---|---|
| Bkg 1 | 2:31 | 2:32 | 2:31 | | | | | |
| Imp B | 3:50 | 3:57 | 3:52 | 1.9 | 29.10 | 0.01 | N/A | 0:07 |
| Bkg 2 | 4:05 | 4:06 | 4:05 | | | | | |
| Bkg 3 | 4:16 | 4:17 | 4:16 | | | | | |
| Region 1 | 4:17 | 4:22 | 4:18 | 0.1 | 1.17 | 0.00 | N/A | 0:05 |
| Bkg 4 | 4:22 | 4:23 | 4:22 | | | | | |
| FMZ | 4:30 | 4:54 | 4:43 | 4.5 | 69.74 | 0.02 | N/A | 0:22 |
| Bkg 5 | 5:27 | 5:28 | 5:27 | | | | | |
| 3 Peaks | | | | 6.4 | 100.00 | 0.03 | | |

Total Area: 25203.8 mAU
Average Background: N/A mAU

Activity of 10 µl sample = 3.72 MBq ; FMZ content in sample = 1.193052 x 10 $^{-5}$ µmol ; OHMZ(impurity B) in sample = 5.459036 x 10 $^{-6}$ µmol
Effective molar activity = 3.72 x 10 $^{-3}$ GBq/1.7389556 x 10 $^{-5}$ µmol = **214 GBq/µmol**

## 2. In-vitro studies

### 2.1. Dose response curves

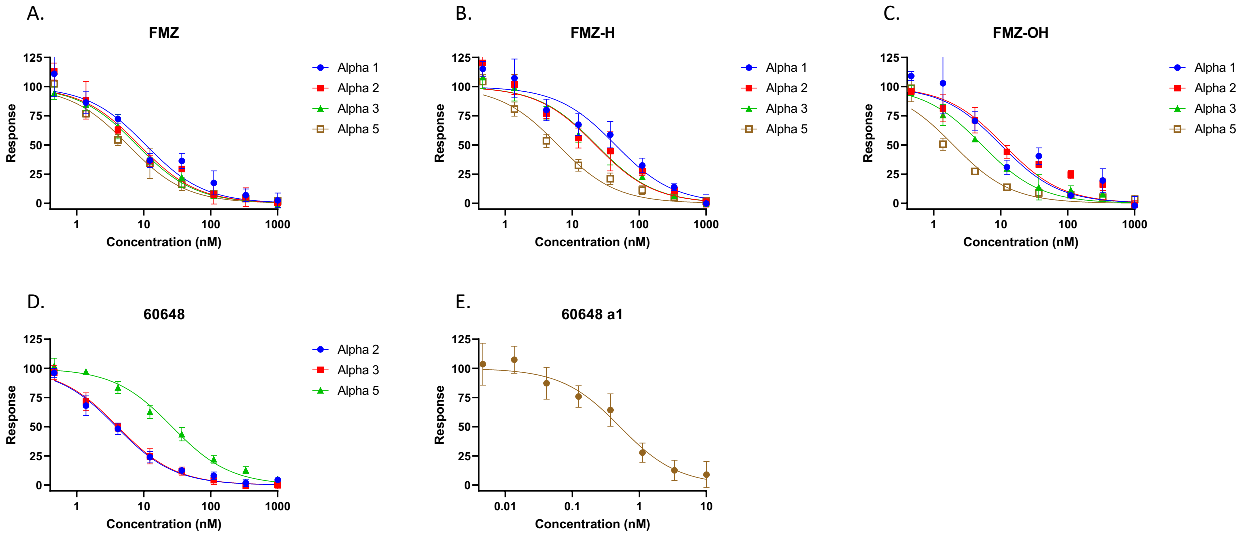


**Figure S9.** Dose response curve of FMZ (A), FMZ-H (B), FMZ-OH (C) affinity for α1-, α2-, α3- and α5β3γ2 subtypes. A top concentration of 1µM was used for all compounds with 40µg per well (α1β3γ2) or 20µg per well (α2-, α3- and α5β3γ2) membrane protein, in competition with 4nM [$^3$H]flumazenil to obtain Ki values for GABAA subtypes. PF-06372865 was used as a reference compound at a top concentration of 1µM for α2-, α3- and α5β3γ2 (D) and 10nM for α1β3γ2 (E) (*n* = 4).

### 3. Semi-preparative HPLC traces

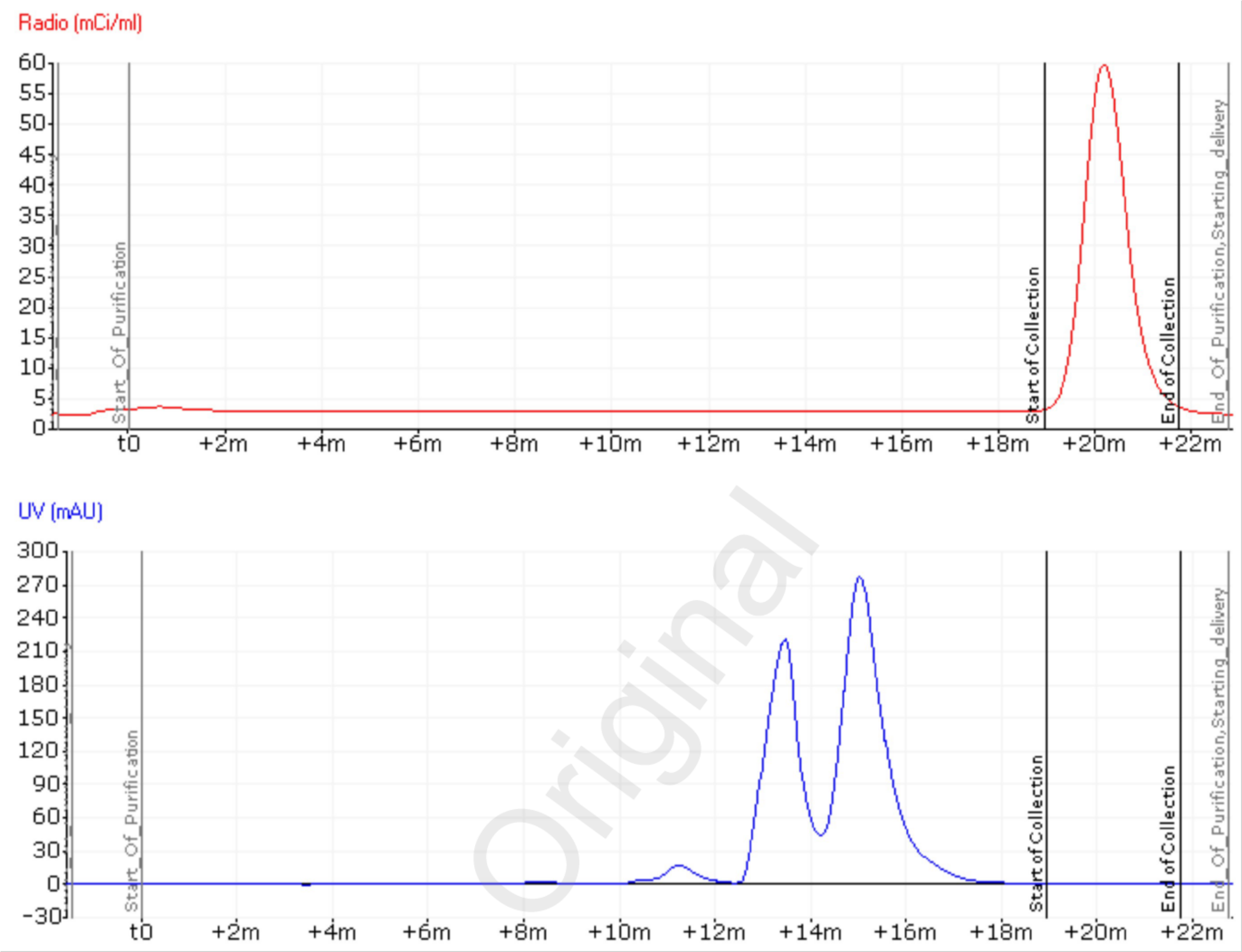


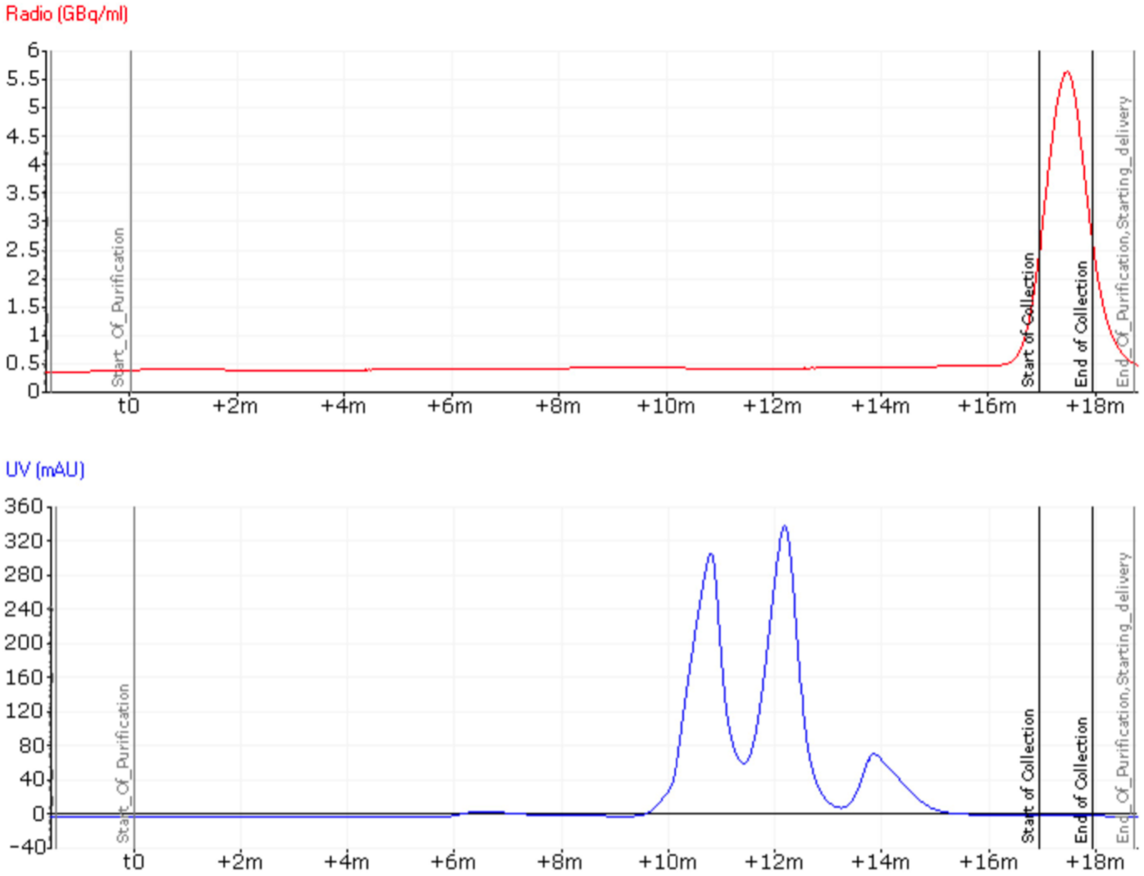

**Figure S10.** Typical semi-preparative HPLC chromatogram obtained during the purification of [$^{18}$F]FMZ under optimized conditions (Top: Trasis; Bottom: Cardiff). The red chromatogram corresponds to radiation detection, whereas the blue chromatogram corresponds to UV detection

## 4. References


(1) Morelle, F.; Vas, A. Fast, Reliable and Universal Spot Test for the Detection of TBA+ in Radiopharmaceutical Preparations. *Eur J Nucl Med Mol Imaging* **2020**, *47* (S1), S682.

(2) Cardinale, C.; Martin, R, Remde, Y *et al*. Procedures for the GMP-compliant Production and Quality Control of [$^{18}$F]PSMA-1007: A Next Generation Radiofluorinated Tracer for the Detection of Prostate Cancer. *Pharmaceuticals* **2017**, *10*, 77.

(3) Gu, Z-Q, Wong, G, Dominguez C, *et al*, Synthesis and Evaluation of Imidazo[1,5-*a*][1,4]benzodiazepine Esters with High Affinities and Selectivities at "Diazepam-Insensitive" Benzodiazepine Receptors. *J. Med. Chem.* **1993**, *36*, 1001-1006.
